# Contrasting influences of ectomycorrhizal fungi on decomposition depending on soil fertility

**DOI:** 10.1111/nph.71567

**Published:** 2026-09-07

**Authors:** Björn D. Lindahl, Karina E. Clemmensen, Christopher W. Fernandez, Peter G. Kennedy, Mathias Mayer, Thomas C. Parker

**Affiliations:** ^1^ Department of Soil and Environment Swedish University of Agricultural Sciences Box 7014 SE‐750 07 Uppsala Sweden; ^2^ Department of Forest Mycology and Plant Pathology Swedish University of Agricultural Sciences Box 7026 SE‐750 07 Uppsala Sweden; ^3^ Department of Biology Syracuse University Syracuse NY 13210 USA; ^4^ Department of Plant & Microbial Biology University of Minnesota St Paul MN 55108 USA; ^5^ Department of Ecosystem Management, Climate and Biodiversity Institute of Forest Ecology, BOKU University Peter Jordan‐Straße 82 1190 Vienna Austria; ^6^ Forest and Soil Ecology Swiss Federal Institute for Forest, Snow and Landscape Research (WSL) Zürcherstrasse 111 8903 Birmensdorf Switzerland; ^7^ Ecological Sciences The James Hutton Institute AB15 8QH Aberdeen UK

**Keywords:** decomposition, forest, Gadgil effect, mycorrhizal guilds, plant–soil feedback, priming, saprotroph

## Abstract

Despite their dominance in many terrestrial ecosystems, the impact of ectomycorrhizal fungi on organic matter decomposition is notably variable across different ecological settings. Here we outline how the influence of ectomycorrhizal fungi on decomposition of belowground organic matter pools depends, not only on the traits of the ectomycorrhizal fungi, but also on the relative dominance of other interacting fungal guilds, in a manner that is predictable across wide gradients in soil fertility. Specifically, we propose that the dominant effect of ectomycorrhizal fungi shifts from ‘priming’ of saprotrophs in the most fertile soils, to inhibition of saprotrophs when conditions become nitrogen‐limited, and to active ‘mining’ of organic matter when nutrient availability is severely constrained. Thus, ectomycorrhizal fungi retard decomposition of organic matter at intermediate soil fertility but accelerate decomposition both in the most fertile and the least fertile soils. These ideas may be applied both on local fertility gradients and at the global biome scale, helping to reconcile inconsistent relationships between ectomycorrhizal fungi and soil organic matter dynamics.

## Graphical abstract

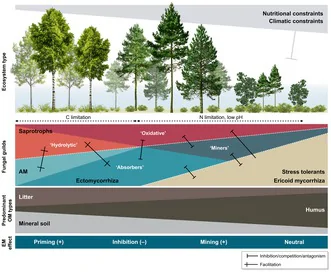

**Table:** 

	Contents	
	Summary	1028
I.	Introduction	1028
II.	Priming in fertile soils	1029
III.	Inhibition as fertility declines	1030
IV.	Nitrogen mining on infertile soils	1030
V.	Conclusions	1032
	References	1033

## I. Introduction

The dynamics of soil organic matter are regulated by an interplay between biological and environmental controls (Schmidt *et al*., 2011). On the biological side, there are striking and globally important connections between the dominant mycorrhizal types of vegetation and belowground organic matter accumulation (Read, 1991; Steidinger *et al*., 2019). Yet, empirical evidence for the regulatory roles of mycorrhizal fungi in decomposition is often contradictory, and different mechanisms appear to be operating depending on ecological context (Frey, 2019; Choreño‐Parra & Treseder, 2024; Pena *et al*., 2025). While dominance by arbuscular mycorrhizal plants has consistently been linked to an inorganic nutrient economy, dominance by ectomycorrhizal and ericoid mycorrhizal plants facilitates mobilisation of organic nutrients (Phillips *et al*., 2013). Ectomycorrhizal‐dominated ecosystems show wide variation in terms of climate, soil types, and vegetation (Read & Perez‐Moreno, 2003), and the ecological settings in which ectomycorrhizal fungi interact with other fungal guilds to regulate organic matter turnover diverge accordingly. Moreover, unlike the more phylogenetically constrained arbuscular mycorrhizal fungi, the ectomycorrhizal symbiosis has evolved independently across multiple fungal lineages (Kohler *et al*., 2015), resulting in wide variation in enzymatic capabilities, mycelial exploration strategies, and modes of soil resource mobilisation (Jörgensen *et al*., 2025).

Fungi have evolved distinct capabilities to decompose organic matter of varying qualities (Table 1). Whereas hydrolases that depolymerise polysaccharides and polypeptides are ubiquitous among most fungal groups (except arbuscular mycorrhizal fungi) and common among prokaryotes, potent oxidative mechanisms required to degrade phenolic compounds, such as lignin, tannin, and melanin, have evolved primarily among Agaricomycetes (Floudas *et al*., 2012). Thus, Agaricomycetes are generally better equipped than other fungi to decompose recalcitrant substrates, including wood, tannin‐rich litter and melanin‐rich mycelial necromass. This extensive ability to degrade plant litter was largely lost, as ectomycorrhizal fungal lineages evolved from saprotrophic agaricomycete ancestors, although most ectomycorrhizal fungi can acquire organic nutrients from easily hydrolysed proteins and microbial necromass (Auer *et al*., 2024). However, some ectomycorrhizal fungal lineages have retained the potent oxidative enzyme systems of their saprotrophic ancestors, and these mycorrhizal decomposers play a critical role in severely nitrogen‐limited ecosystems, liberating nutrients from recalcitrant pools (Lindahl & Tunlid, 2015; Barbi *et al*., 2026). Recently, ectomycorrhizal fungi were proposed to vary along a trait spectrum, from ‘absorbers’, with rapidly growing, hydrophilic mycelium and low decomposer capacity, to ‘miners’, with slowly growing, hydrophobic mycelium and high decomposer capacity (Table 1; Jörgensen *et al*., 2025; Cox, 2026). This spectrum partly overlaps with the exploration types proposed by Agerer (2001), which are frequently used as an integrating ectomycorrhizal fungal trait related to organic matter dynamics (e.g. Khalfallah *et al*., 2024).

**Table 1:** Here we outline six fungal guilds in order of increasing decomposer capabilities and provide examples of important fungal groups (focusing on common soil fungi).

Guild	Decomposer capabilities	Examples of taxonomic groups
Arbuscular mycorrhizal fungi	Little capacity to depolymerise organic resources	Glomeromycota
Ectomycorrhizal absorbers	Take up soluble nitrogen and hydrolyse proteins and microbial necromass, but cannot access tannin‐bound or melanised resources	Atheliales,^a^ Thelephoraceae, *Amanita*, *Clavulina*, *Hygrophorus*, *Inocybe*, *Laccaria*, *Tricholoma*, *Cenococcum*, *Elaphomyces*
Hydrolytic saprotrophs	Hydrolyse polysaccharides and proteins, but cannot oxidise lignified tissues or use tannin‐bound or melanised resources	Mucoromycota, Dothidiomycetes, Sordariomycetes, Saccharomycetes, Microbotryomycetes, Eurotiales, Tremellales
Ericoid mycorrhizal fungi and other stress tolerants	Have versatile hydrolytic capabilities and oxidative phenol oxidases, but lack peroxidases	Leotiomycetes, Chaetothyriales, Sebacinales
Ectomycorrhizal miners	Can access most organic N, using peroxidases or Fenton reactions	Cortinariaceae, some Russulaceae, *Hebeloma*, *Hysterangium*, *Paxillus*, Bankeraceae, *Gautieria*, *Piloderma* ^a^
Saprotrophic Agaricomycetes	Can fully decompose most organic substrates, including lignified litter	*Mycena*, *Gallerina*, *Gymnopus*, and other Agaricales, Trechisporales, Cantharellales

^a^ The degrading capacity of *Piloderma* species (Atheliales) remains uncertain.

Ericoid mycorrhizal fungi have versatile hydrolytic capabilities and may produce a variety of enzymes targeting plant cell wall polysaccharides, potentially allowing them to shift between biotrophic symbiosis and free‐living saprotrophic strategies (Martino *et al*., 2018). In an influential paper, Read & Perez‐Moreno (2003) suggested that the capacity to exploit organic nutrient pools is more extensively developed in ericoid mycorrhizal fungi than in ectomycorrhizal fungi. However, they also acknowledged the large variation among ectomycorrhizal fungal groups, which has been increasingly highlighted by more recent research (Zak *et al*., 2019). Thus, while ericoid mycorrhizal fungi are more versatile decomposers than ectomycorrhizal fungi with distinct ‘absorber’ traits, they lack the potent oxidative mechanisms that facilitate significant losses of more recalcitrant organic matter stocks (Miyauchi *et al*., 2020). With their retained decomposer capabilities, ectomycorrhizal fungal ‘miners’ seem superior to ericoid mycorrhizal fungi and other stress‐tolerant Ascomycetes in causing significant mass loss of organic humus layers (Lindahl *et al*., 2021; Mielke *et al*., 2025).

Here we highlight how the influence of ectomycorrhizal fungi on soil organic matter pools depends, not only on the traits of the ectomycorrhizal fungi themselves, but also on the traits of other fungal guilds with which they interact. We further propose that interactions among fungal guilds are strongly shaped by soil fertility (i.e. pH, water availability, texture, mineralogy, weathering rates, and nutrient and carbon availability through turnover of organic matter), resulting in contrasting effects of ectomycorrhizal fungi on belowground organic matter dynamics.

## II. Priming in fertile soils

Ectomycorrhizal fungi can facilitate the transfer of recently assimilated plant carbon from roots into the surrounding soil, where labile carbon compounds may stimulate free‐living saprotrophs and enhance decomposition processes (Frey, 2019). Such root‐dependent stimulation of saprotrophic decomposition is generally referred to as rhizosphere (or mycorrhizosphere) priming (Huo *et al*., 2017) and may accelerate litter mass loss at the soil surface (Subke *et al*., 2011; Lang *et al*., 2021) as well as turnover of older organic matter in mineral soils (Brzostek *et al*., 2015; Mayer *et al*., 2023), thus constraining carbon accumulation. Priming can be further enhanced by inputs of easily decomposable hyphal necromass to the soil (Zhang *et al*., 2019).

On fertile soils where plant available nutrients are abundant and net primary production is high, carbon‐limited hydrolytic saprotrophic fungi and bacteria dominate microbial communities and decomposition (Bahnmann *et al*., 2018; Mayer *et al*., 2021; Yu *et al*., 2022), and relatively high litter quality and soil pH promote rapid litter decay (Prescott & Vesterdal, 2021). This process is further enhanced by active soil fauna, such as earthworms, which fragment and translocate organic material into deeper soil horizons (Barthod *et al*., 2020). Consequently, the accumulation of an organic layer is constrained, and the stabilisation of soil organic matter is primarily governed by associations with mineral surfaces and incorporation into soil aggregates (Lehmann & Kleber, 2015; Prescott & Vesterdal, 2021). Fast nitrogen mineralisation favours arbuscular mycorrhizal plants (Phillips *et al*., 2013) and selects for nitrophilic ‘absorbers’ in the ectomycorrhizal fungal community (Pena & Tibbett, 2024; Cox, 2026). Under these conditions, the priming effect of ectomycorrhizal fungi likely plays a dominant ecological role (Choreño‐Parra & Treseder, 2024). For example, in ectomycorrhizal fungal communities dominated by Thelephorales, these fungi likely contributed substantially to hyphal transfer of plant‐derived carbon to soil bacteria (Gorka *et al*., 2019). Further, in a temperate *Fagus* forest on fertile soil, tree girdling decreased the abundance of ectomycorrhizal fungi (notably *Clavulina* spp.), decreased the abundance of hydrolytic saprotrophic fungi (mainly *Mortierella* and *Trichoderma* spp.), and reduced decomposition (Mayer *et al*., 2023), suggesting disrupted ectomycorrhizal fungal priming.

## III. Inhibition as fertility declines

As soil fertility declines, net primary production and the quality of plant litter generally decrease as the contents of lignin and tannin increase (Cornwell *et al*., 2008). In parallel, as soils become more acidic, activities of soil fauna and bacteria are reduced, and litter and organic humus layers accumulate on top of the mineral soil (Prescott & Vesterdal, 2021). As bacterial and fungal saprotrophs are increasingly constrained by low nitrogen availability, the stimulatory priming effect of carbon‐rich mycorrhizal exudates declines. In addition, decreasing quality of fungal mycelium (e.g. high melanin content and C : N ratios) may turn necromass priming into inhibition of decomposition (Fernandez & Koide, 2014; DeLancey *et al*., 2024; Carrillo *et al*., 2025). Decomposition depends increasingly on saprotrophic Agaricomycetes with potent oxidative capabilities, and low nitrogen mineralisation reduces the abundance of arbuscular mycorrhizal plants. At intermediate soil fertility, ectomycorrhizal fungal communities typically remain dominated by ‘absorbers’ with low decomposer capabilities. These ectomycorrhizal fungi compete with the more potent saprotrophic Agaricomycetes for limiting nitrogen, hampering their activity as well as overall decomposition in line with the ‘Gadgil hypothesis’ (Gadgil & Gadgil, 1971; Fernandez & Kennedy, 2016). The magnitude of this effect appears to increase as litter quality and nutrient availability decrease (Kyaschenko *et al*., 2017; Smith & Wan, 2019; Fernandez *et al*., 2020; Mayer *et al*., 2023). Importantly, soil pH may modulate the strength of this competitive effect by altering organic nitrogen availability to ectomycorrhizal fungi and saprotrophs. Specifically, as pH increases (*c*. > 5), organic nitrogen bound to polyphenol complexes dissociates and becomes more accessible to hydrolytic attack. This greater availability may enable ‘absorber’ ectomycorrhizal fungi to compete more directly with saprotrophs for the released nitrogen pool, potentially intensifying the Gadgil suppression of organic matter decomposition (Fernandez & See, 2025). For example, in a temperate *Pinus* forest, hampering of roots by trenching suppressed the ectomycorrhizal fungal community (particularly *Thelephora* spp.), increased nitrogen availability, and stimulated fungal saprotrophs (e.g. *Mortierella* and *Trechispora* spp.) as well as leaf litter decomposition rates (Fernandez *et al*., 2020). In ecosystems with a pronounced Gadgil suppression, organic matter decomposition and accumulation depend on the ectomycorrhizal to saprotrophic fungal ratios, with slower decomposition and higher soil carbon accumulation at high ratios (DeLancey *et al*., 2024).

## IV. Nitrogen mining on infertile soils

On less fertile soils, saprotrophic decomposers are increasingly constrained by low pH and low nutrient availability (Lindahl *et al*., 2021), with most nitrogen tightly bound in recalcitrant organic complexes (Fernandez & See, 2025). In parallel, stress‐tolerant ericaceous dwarf shrubs and mosses become dominant in the forest understory, which further deteriorates litter quality (Cornelissen *et al*., 2001) and suppresses decomposers through chemical antagonism and competition from ericoid mycorrhizal fungi (Fanin *et al*., 2022; Mielke *et al*., 2025). As a result, the ectomycorrhizal fungal community shifts in favour of species with more prominent oxidative decomposer capacities – organic matter ‘miners’ (Pellitier & Zak, 2021; Argiroff *et al*., 2022; Packard *et al*., 2025). These species confine saprotrophs to the uppermost layer of fresh plant litter and reinforce the Gadgil suppression in the litter layer, whereas the more decomposed humus is dominated by ectomycorrhizal fungi and stress‐tolerant Ascomycetes. In this setting, the ectomycorrhizal fungal miners stand out as the principal decomposers in the humus (Clemmensen *et al*., 2015; Barbi *et al*., 2026). For example, in a boreal *Picea/Pinus* forest, root severing by trenching largely eliminated the ectomycorrhizal fungal community (12% of which were *Cortinarius* species). This stimulated litter mass loss rates, indicating Gadgil suppression of early stages of decomposition, but at the same time the loss of ectomycorrhizal fungal ‘miners’ led to a 91% reduction of Mn‐peroxidase activity in the humus layer (Sterkenburg *et al*., 2018). Further, across a fertility gradient of coniferous forests in northern Sweden, saprotrophic Agaricomycetes (mainly *Mycena* spp.) decreased in abundance with declining pH (5 => 3), whereas a group of related *Cortinarius* spp. were more common in unfertile forests, and their presence was linked to 33% lower carbon storage in the organic horizon (Lindahl *et al*., 2021). In *Pseudotsuga* stands planted across a natural soil fertility gradient, peroxidase activities increased whereas carbon stocks in the organic horizon decreased with declining soil fertility, linked to increasing abundance of *Piloderma* spp. in the ectomycorrhizal community (Kranabetter *et al*., 2025). Notably, the importance of ectomycorrhizal fungi as decomposers has been proposed to be conditional on an ericaceous understory (Ward *et al*., 2021) and low pH (Fernandez & See, 2025).

In the least fertile boreal forests, as well as in alpine, arctic, and heathland ecosystems, plants and soil fungi are under severe stress from nutrient limitation, acidic conditions, low temperatures, short growing seasons and, sometimes, soil anoxia. In these environments, ericoid dwarf shrubs and non‐mycorrhizal sedges take over and deploy diverse nutrient acquisition strategies to facilitate their slow growth (McKane *et al*., 2002). Ericoid mycorrhizal fungi can mobilise limited amounts of organic‐bound nitrogen to meet the low demand from hosts, and their versatile decomposer capabilities may enable them to play dual roles as both plant mutualistic symbionts and free‐living degraders of organic matter (Martino *et al*., 2018) throughout seasonal cycles. However, they lack potent oxidative enzymes, and when the primary production of ectomycorrhizal hosts declines, and competition from ericoid mycorrhizal fungi intensifies (Fanin *et al*., 2022; Mielke *et al*., 2025), ectomycorrhizal ‘miners’ may lack the energy required to maintain organic matter oxidation (Clemmensen *et al*., 2015; Packard *et al*., 2025). Low availability of manganese, required for the activity of manganese peroxidases, may put additional constraints on oxidative decomposition in ectomycorrhizal forests (Zhang *et al*., 2024). When more productive shrubs/trees establish in ericoid‐dominated ecosystems, increased photosynthesis allows for higher carbon allocation to ectomycorrhizal ‘miners’, and increased decomposition and nutrient mobilisation could cause a ‘tipping point’ associated with significant losses of organic matter (Clemmensen *et al*., 2015; Parker *et al*., 2015, 2021; Defrenne *et al*., 2023). For example, in a sub‐arctic *Betula* forest with soil fungal communities dominated by stress‐tolerant Helotiales, Chaetothyriales and Sebacinales spp., elimination of ectomycorrhizal fungi (44% of which were *Cortinarius* species) decreased decomposition of organic substrates. However, above the forest line in the alpine heath, ‘miners’ were more rare in the ectomycorrhizal fungal community (7% *Cortinarius* species), and large amounts of organic matter accumulated belowground, despite low input rates (Clemmensen *et al*., 2021).

## V. Conclusions

Altogether, the contrasting interactions in different ecological contexts imply that the role of ectomycorrhizal fungi as regulators of organic matter dynamics shifts along soil fertility gradients (Fig. 1). Such gradients may either be local, depending on different hydrology, disturbances, soil types, bedrock, and elevation, or at the biome scale, depending on macro‐climatic factors. In the most fertile ectomycorrhizal‐dominated ecosystems (typically lowland deciduous forests), ectomycorrhizal fungi facilitate decomposition by priming of hydrolytic saprotrophic fungi and bacteria. When ecosystem fertility and organic matter quality decrease (such as in mixed and coniferous temperate forests), more potent Agaricomycetes are required to maintain decomposition, and less potent ectomycorrhizal fungal ‘absorbers’ may suppress decomposition by competing for limiting nutrients, in line with the Gadgil hypothesis. Even further down the fertility gradient (e.g. in many boreal forests), saprotrophs are restricted and decomposition depends primarily on ectomycorrhizal fungal ‘miners’, which drive decomposition in competition with the less potent ericoid mycorrhizal fungi. In alpine and arctic conditions, neither saprotrophic Agaricomycetes nor ectomycorrhizal fungal miners thrive, and the absence of efficient decomposers may lead to slow decomposition and accumulation of vast organic matter pools, despite low primary production. We, thus, propose that ectomycorrhizal fungi accelerate decomposition at both ends of the fertility gradient but suppress decomposition at intermediate fertility. This hypothesis may be tested by a series of standardised field experiments along fertility gradients, which may be either local or more geographically dispersed and climate‐related. In such experiments, the influence of ectomycorrhizal fungi could be disrupted by tree girdling or root severing (trenching), after which decomposition rates could be estimated using mesh bags filled with organic substrates or based on long‐term changes in organic matter pools. A challenge is to account for treatment‐induced alterations of inputs (roots and mycorrhizal mycelium). Indirect assays, such as enzyme activity analyses and litter chemical analyses may complement direct measurements of organic matter losses, and metatranscriptomic analyses of gene expression may be used to test hypotheses about mechanisms.

**Fig. 1:**
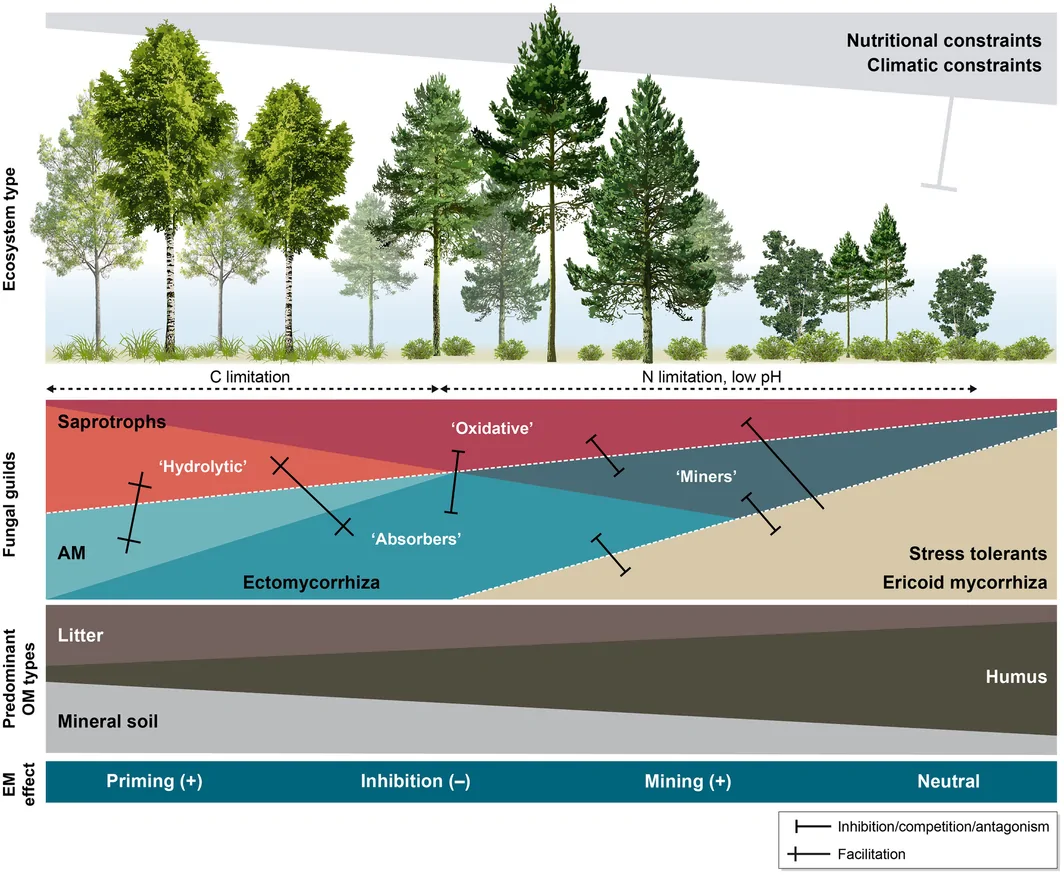
Ecosystem types along a gradient of increasing nutritional and climatic constraints with an overall transition from carbon (C)‐ to nitrogen (N)‐limitation on decomposition. Soil organic matter (OM) types are portrayed by the relative dominance of litter, humus or mineral soil (rather than total stocks). Along this gradient, the soil fungal community transitions from a dominance of saprotrophic (red) and mycorrhizal fungal guilds (blue) towards dominance of stress‐tolerant and ericoid mycorrhizal fungi (light brown). Positive (+) and negative (−) interactions between fungal guilds are indicated by black symbols. Within the saprotrophic guild, fungi relying mainly on hydrolytic enzymes dominate when litter quality is high, decomposition is rapid and C limitation prevails. Here, soil organic matter (OM) is predominantly in the form of relatively fresh litter or mineral associated organic matter. As N limitation intensifies and litter quality declines, fungi with oxidative capacities increase in abundance. Within the mycorrhizal guilds, in the most fertile systems, arbuscular mycorrhizal (AM) fungi and ectomycorrhizal ‘absorbers’ benefit from mineralised nutrients and facilitate saprotrophs by transporting C into the soil. In less fertile systems, the ectomycorrhizal ‘absorbers’ instead compete with saprotrophs for mineral and hydrolysable N compounds. As N is increasingly bound in the organic humus layer, pH decreases, and ericoid plants increase in abundance, ectomycorrhizal ‘miners’ with oxidative capacities take over as the principal decomposers. The most climatically constrained systems are dominated by stress tolerants, with potentially versatile saprotrophic/biotrophic lifestyles, but without prominent oxidative decomposer capacities (leading to humus accumulation). Thus, moving from high to low soil fertility, the net ectomycorrhizal (EM) effect on organic matter decomposition changes from ‘priming’, that is, stimulation, of free‐living saprotrophic decomposers in the most fertile soils, via ‘inhibition’ of saprotrophic activities due to competition for limiting N, that is, a Gadgil effect, to ‘mining’, that is, direct decomposition of organic matter where nitrogen is more strongly limiting, and finally neutral in the least fertile ecosystems, where ectomycorrhizal fungi play a subordinate role.

Although Gadgil suppression has been proposed as a global driver of belowground organic matter accumulation in ectomycorrhizal ecosystems (Averill *et al*., 2014), we propose that this positive ectomycorrhizal effect on carbon sequestration is conditional on intermediate levels of ecosystem fertility and not valid in too rich soils (no N limitation of decomposition) or too poor soils (ectomycorrhizal miners drive decomposition). Incorporation of this context dependency may provide a more nuanced understanding of how ectomycorrhizal fungi interplay with soil carbon dynamics and help to explain why ectomycorrhizal fungi correlate positively with belowground carbon pools on the largest scales (Steidinger *et al*., 2019) yet often appear to stimulate organic matter turnover in local studies (Choreño‐Parra & Treseder, 2024; DeLancey *et al*., 2025). We conclude that ectomycorrhizal interactions are decisive for the biological regulation of organic matter stability in temperate and boreal ecosystems. Biological and environmental constraints on decomposition should be evaluated together with organic inputs (amounts and qualities) for a better understanding and management of ecosystems as carbon sinks.

## Competing interests

None declared.

## Author contributions

BDL initiated the project. BDL, KEC, CWF, PGK, MM and TCP all participated in the writing of the paper.

## Disclaimer

The New Phytologist Foundation remains neutral with regard to jurisdictional claims in maps and in any institutional affiliations.
